# Rapid detection and quantification of glyphosate in water using a handheld portable biosensor

**DOI:** 10.1038/s41598-026-44827-4

**Published:** 2026-04-11

**Authors:** Andreea Stroia, Boon Chong Cheah, Samadhan B. Patil, Valerio F. Annese, Reynard Spiess, Christoph Busche, David R. S. Cumming, Michael P. Barrett, Dharmendra S. Dheeman

**Affiliations:** 1 Wellcome Centre for Integrative Parasitology, Institute of Infection, Immunity and Inflammation, University of Glasgow, Glasgow, G12 8TA UK; 2 Electronics and Nanoscale Engineering, James Watt School of Engineering, University of Glasgow, Glasgow, G12 8LT UK; 3 School of Physics, Engineering and Technology, University of York, Heslington, York, YO10 5DD UK; 4 York Biomedical Research Institute, University of York, Heslington, York, YO10 5NG UK; 5 Manchester Institute of Biotechnology, School of Chemistry, University of Manchester, 131 Princes Street, Manchester, M1 7DN UK; 6 School of Chemistry, University of Glasgow, Glasgow, G12 8QQ UK; 7 Present Address: ASML Veldhoven, De Run 6501, Veldhoven, 5504 DR The Netherlands; 8 Present Address: Center for Nano Science and Technology, Istituto Italiano di Tecnologia, Milan, 20134 Italy

**Keywords:** Glyphosate, herbicide, glyphosate *N*-acetyltransferase, lab-on-a-chip, CMOS sensor, photodiode array, Biological techniques, Chemistry, Environmental sciences

## Abstract

Glyphosate is the most extensively applied systemic herbicide worldwide, yet its safety remains under scrutiny, with ongoing investigations into potential carcinogenicity. Epidemiological studies associate chronic glyphosate exposure with elevated risks of non-Hodgkin lymphoma and possible endocrine disruption, emphasizing the need for sensitive detection methods. Here, we report a handheld enzymatic biosensor, GlyphoSense Chip, for direct, rapid detection of underivatized glyphosate in drinking water. The device integrates a photodiode-based CMOS chip with an engineered glyphosate *N*-acetyltransferase and a colorimetric reaction, achieving a sensitivity of 38 µV·mL µg^−1^·s^−1^ and quantification within one minute. Biosensor response was linear over 0.016–12.5 µg mL^−1^ (*R*^2^ = 0.993) with a detection limit of 0.028 µg mL^−1^. Recovery analysis in fortified tap water yielded relative standard errors of 1.2–5.8%, and results were statistically indistinguishable from quantitative mass spectrometry (*p* > 0.05). This work establishes a robust, field-deployable platform for glyphosate monitoring in water resource safety applications.

**Supplementary Information:**

The online version contains supplementary material available at 10.1038/s41598-026-44827-4.

## Introduction

Glyphosate is one of the most widely used herbicides worldwide, acting by specifically targeting the shikimate pathway in plants. This pathway is responsible for the biosynthesis of aromatic amino acids, tryptophan, tyrosine, and phenylalanine, as well as a variety of other key metabolites derived from chorismate^1,2^. Glyphosate exerts its herbicidal activity by binding tightly to 5-enolpyruvylshikimate-3-phosphate synthase (EPSPS), thereby blocking the conversion of shikimate-3-phosphate to 5-enolpyruvylshikimate-3-phosphate^3^. Since mammals lack the shikimate pathway, glyphosate was initially considered safe for humans and animals. However, over the past decade, mounting evidence has challenged this assumption. In 2015, the World Health Organization (WHO) initiated a re-evaluation of glyphosate following concerns over its toxicity^4–7^. That same year, the International Agency for Research on Cancer (IARC) classified glyphosate as a “probable human carcinogen” based on strong evidence of carcinogenicity in laboratory animals^8^.

Regulatory guidelines for glyphosate levels in water vary widely across the globe. In the United States, the Environmental Protection Agency (EPA; https://www.epa.gov/) permits a maximum contaminant level (MCL) of 0.7 µg mL^−1^ in drinking water. Whereas, the European Union enforces far stricter limits: the Drinking Water Directive caps pesticide concentrations at just 0.001 µg mL^−1^^9,10^. These differences highlight both the global concern surrounding glyphosate exposure and the challenges in setting consistent safety thresholds.

From a chemical perspective, glyphosate contains a phosphonate group (R–PO(OH)₂) that forms strong complexes with metal ions such as Fe^3+^, Al^3+^, Ca^2+^, and Mg^2+^. This property complicates its breakdown in soil and water, where glyphosate is primarily degraded by soil microbes to yield AMPA as a major product that is often more persistent (with half-lives up to 2–3 times longer) and mobile, allowing these residues to accumulate in the food chain and pose potential risks to wildlife and livestock through chronic exposure, making its environmental fate more complex than once believed^11,12^. Detecting glyphosate in environmental samples is also notoriously difficult due to its high polarity and structural similarity to amino acids^13^. Traditional detection techniques, including high-performance liquid chromatography (HPLC) with derivatization, gas chromatography-mass spectrometry (GC-MS), and nitrogen-phosphorus detection, offer excellent sensitivity but require labor-intensive sample preparation, costly equipment, and trained personnel^14–17^. These limitations underscore the urgent need for rapid, affordable, and field-deployable detection methods that can be operated by non-specialists.

Against this backdrop, biosensors are gaining prominence as promising alternatives for rapid, cost-effective, and user-friendly detection of contaminants. Portable biosensing devices have already demonstrated their utility in diverse biomedical and environmental applications, due to their miniaturization, and potential for high sensitivity and selectivity^18–25^. In particular, complementary metal–oxide–semiconductor (CMOS) technology provides an attractive foundation for integrating biological sensing elements with scalable electronic platforms. CMOS-based photodiode (PD) arrays are inexpensive, compact, and compatible with portable device fabrication. When coupled with selective enzymatic reactions, they enable highly sensitive and specific detection of analytes^26^. Enzymes remain the most widely employed biorecognition elements in biosensors due to their intrinsic catalytic efficiency and substrate specificity, offering natural molecular recognition unmatched by many synthetic alternatives^27–30^.

Here we introduce a compact GlyphoSense Chip (CMOS-based optoelectronic sensor) capable of detecting and quantifying glyphosate in contaminated water samples within one minute which meets the requirements of regulatory agencies in the USA and Canada, as well as detecting elevated glyphosate concentrations in water for European regulatory agencies. The detection is leveraged by the use of a unique genetically engineered glyphosate-specific *N*-acetyltransferase (GAT) from *Bacillus licheniformis* coupled to a colorimetric reaction that enables direct detection of underivatized glyphosate presence in water samples. The resulting change in absorbance is captured in real-time by the GlyphoSense Chip. The GlyphoSense Chip is designed as a handheld unit with USB connectivity to a custom-built user interface, enabling rapid visualization, capture, and analysis of data. This device platform offers a practical, field-deployable solution for monitoring glyphosate contamination in environmental water samples.

## Materials and methods

All reagents were purchased from Sigma-Aldrich Co. Ltd (Dorset, UK) at the highest available purity-levels unless specified otherwise.

### GlyphoSense chip

**Fig. 1:**
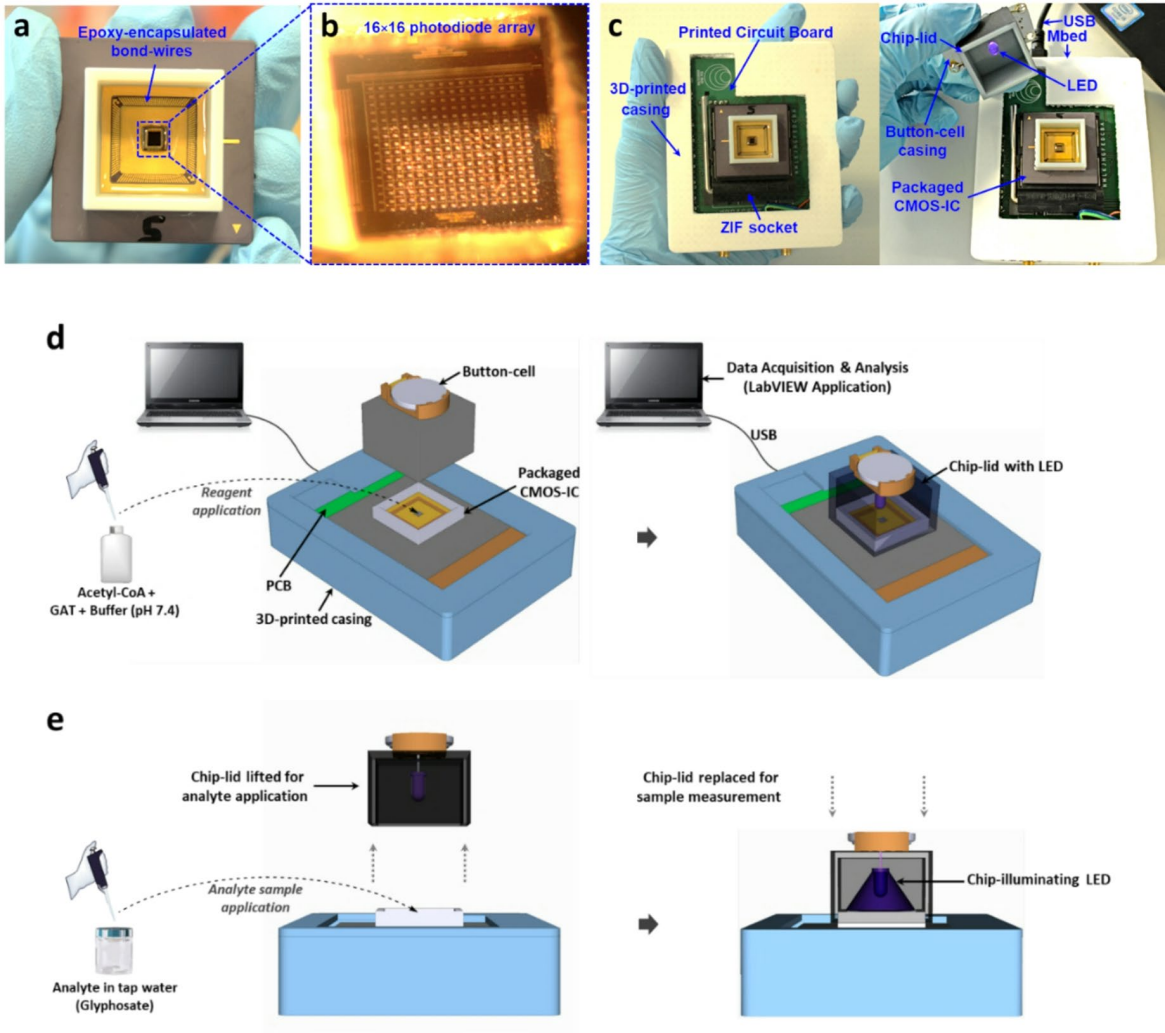
GlyphoSense Chip: design and measurement workflow. (**a**) Packaged CMOS integrated circuit bonded to a pin grid array (PGA)-144 carrier. (**b**) Micrograph of the fabricated CMOS chip with a 16 × 16 photodiode array and wire-bond connections. (**c**) CMOS chip mounted on a test PCB via ZIF-socket and enclosed in a 3D-printed sensor body with a light-proof lid housing an LED for illumination. The PCB provides power, control circuitry, and PC (LabVIEW) interfacing. (**d**) Reaction reagents applied to the CMOS chip, followed by lid placement with a button-cell powered LED for baseline recording. (**e**) Lid briefly lifted for analyte addition, and the lid is then replaced to measure the sample response on a PC-based custom-built LabVIEW application.

The GlyphoSense Chip was purpose designed and fabricated through a 0.35 μm CMOS foundry service (ams AG, Austria) and featured a 16 × 16 photodiode array with integrated addressing and readout circuitry. The device measured 3.4 × 3.6 mm overall, with a 1.6 × 1.6 mm active sensing core. Each sensing element occupied 10 × 24 μm (Fig. 1a–b).

To enable liquid-phase assays, the chip was packaged to shield bond wires from fluid exposure using commercial epoxy resins following^26^ The chip was first mounted onto a chip carrier (PGA-144) using epoxy resin (EPO-TEK H74, Epoxy Technologies Inc., USA), cured at 150 °C for 10 min. Pads were wire-bonded to the carrier and subsequently encapsulated with epoxy resin (EPO-TEK 302-3M), while the sensor array was temporarily masked with a ~ 3 mm^2^ PDMS block to prevent resin overflow. After curing at room temperature for 24 h, the PDMS was removed, leaving a cavity above the sensing area.

A custom polypropylene casing was 3D-printed and affixed to the carrier with epoxy resin, cured at 65 °C for 3 h. For use in well-lit environments, a snug-fitting, light-tight lid was also fabricated. An LED (380–410 nm; LUMEX Inc., USA), powered by a 3 V button cell, was integrated into the lid to provide uniform illumination.

The packaged chip was mounted onto a printed circuit board (PCB) via a zero-insertion force (ZIF) socket (Fig. 1c). The PCB was interfaced with a programmable Mbed STM32 Nucleo microcontroller board (STMicroelectronics, UK), which linked the sensor to a data acquisition (DAQ) system. This setup enabled addressing and readout of the sensing array, with data visualization, processing, and analysis performed through a custom PC-based LabVIEW program.

### GAT expression and purification

The *gat* gene from *Bacillus licheniformis* (R11 mutant;^2,31^ was synthesized with codons optimized for *E. coli* expression and cloned into pUC57-Kan (GenScript, Hong Kong). The gene was subcloned into pET15b via NdeI/BamHI, generating an N-terminal His_6_-tag fusion (Fig. S1-S2). The construct was verified by sequencing (Eurofins Genomics, UK) and transformed into *E. coli* BL21(DE3).

Recombinant GAT was purified by immobilized metal affinity chromatography (His GraviTrap, GE Healthcare) at pH 7.4 and ~ 21 °C, followed by buffer exchange and concentration (Amicon Ultra, 10 kDa MWCO, Merck Millipore). The protein was further purified and analyzed by size-exclusion chromatography (Superdex 75 10/300 GL, GE Healthcare; AKTA system) in 20 mM Tris-HCl, 300 mM NaCl, pH 7.4, at 4 °C (Fig. S3).

Purified GAT was supplemented with 10% (v/v) glycerol, aliquoted (25–50 µL), and stored at − 80 °C. Protein concentration was determined at 280 nm using the calculated extinction coefficient (NanoDrop 1000, Thermo Scientific, UK).

### GAT activity assay

GAT activity was initially assayed off-chip by monitoring the reduction of 5,5’-dithio-bis-2-nitrobenzoic acid (DTNB) at 412 nm^32^. Reactions (250 µL) were conducted at 30 °C in a Shimadzu UV-2550 spectrophotometer. The reaction was initiated by adding acetyl-CoA (AcCoA), and activity was calculated using the extinction coefficient of the 2-nitro-5-thiobenzoate anion (ε = 14,150 M^−1^ cm^−1^). Samples were diluted to maintain linear initial rates, with controls lacking AcCoA and/or enzyme.

One unit (U) of activity corresponded to the conversion of 1 µmol of glyphosate to N-acetyl-glyphosate per minute under the assay conditions. Kinetic parameters were determined by varying glyphosate concentrations (4.2–101.4 µg mL^−1^) and fitting the data by non-linear regression (GraphPad Prism 10.4.1).

### Glyphosate samples

A glyphosate stock solution (338 µg mL^−1^) was prepared by dissolving analytical-grade glyphosate powder (PESTANAL^®^, Sigma Aldrich, UK) in deionized water (Milli-Q, Merck Millipore, UK). From this stock, a series of working standards was prepared at concentrations ranging from 0.016 to 101.44 µg mL^−1^ for GlyphoSense Chip calibration and validation. A commercial formulation, Roundup^®^ (UK), supplied as a water-soluble concentrate, was also analyzed. This formulation contains glyphosate isopropylamine salt at 7.2 g L^−1^, equivalent to 9.7 g L^−1^glyphosate acid. To assess matrix effects and recovery, local tap water samples were fortified with either pure glyphosate or the Roundup formulation at concentrations between 1.0 and 8.45 µg mL^−1^. These spiked samples were used alongside standards to evaluate potential interference from the sample matrix and recovery assessment.

### GlyphoSense chip response and calibration

Glyphosate concentrations in water were measured using the GlyphoSense Chip connected to a PC via USB (Fig. 1c). A 250 µL assay mixture, consisting of 0.2 mM acetyl-CoA (AcCoA), 0.32 mM DTNB, 1 mM EDTA, and glyphosate sample in 100 mM Tris-HCl buffer (pH 7.4) was applied to the chip surface and covered with a lid containing an LED. All components, except the reaction initiator GAT (maintained at 4 °C on ice), were equilibrated to ambient temperature (~ 20 °C). Amplitude signals were recorded with a LabVIEW-based application. After establishing a stable baseline, 5 µL (3 U) buffer-diluted GAT was added, gently mixed without introducing air bubbles, and the lid replaced. Signal changes were recorded for 70 s. GlyphoSense Chip response to glyphosate in water was measured over 0.0169–67.63 µg mL^−1^. Initial rates (v₀) were calculated from the slope (∆mV/∆t) of the linear regression (R^2^ ≥ 0.98) of the first 10 s. After each assay, the chip was rinsed with isopropanol and deionized water and blow-dried with compressed air to remove residues before subsequent measurements.

## Results and discussion

### GlyphoSense chip

The GlyphoSense Chip consists of 256 individual pixels (Fig. 1a–c), each capable of simultaneously recording data during sample measurements. This multi-pixel photodiode array minimizes background noise, and data from each measurement can be averaged across all sensor pixels or analyzed on a pixel-by-pixel basis. The chip is paired with a standard button-cell powered light-emitting diode (LED) emitting light at ~ 410 nm (Fig. 1c). A printed circuit board (PCB) connects the GlyphoSense Chip to the system, enabling digitization of the sensor signals and transfer to LabVIEW software for real-time data visualization and processing (Fig. 1c-d). The chip exhibits a responsivity of ~ 5 V mW^−1^ at 410 nm^33^. Detection of glyphosate is achieved through a coupled colorimetric reaction, which shows peak absorption at 410 nm, facilitated by glyphosate acetylation catalyzed by an engineered *N*-acetyltransferase enzyme (Fig. S6). In field applications, water samples suspected of glyphosate contamination can be applied directly to the chip, pre-loaded with assay mixture and GAT, to trigger the biosensor response (Fig. 1d,e).

### Off-chip GAT assays and kinetics

The biological sensing element paired with the GlyphoSense Chip is a genetically evolved R11 mutant of glyphosate *N*-acetyltransferase (GAT), which exhibits enhanced activity toward glyphosate^1,2,31^. We cloned a synthetic, codon-optimized gat gene encoding the R11 mutant with an N-terminal 6×His tag to facilitate optimal expression in *E. coli* (Figs. S1, S2). The expressed GAT enzyme was purified through two consecutive chromatographic steps (Fig. S3) and subsequently used for a series of initial kinetic measurements.

Before conducting measurements on the GlyphoSense Chip, we characterized the enzyme kinetics in solution to determine optimal conditions for GAT activity. Off-chip spectrophotometric assays were performed using varying glyphosate concentrations, with changes in absorbance at 410 nm (ΔA_410nm_·s^−1^) monitored over time (Fig. S4a). Analysis of these data yielded a Michaelis–Menten constant (*K*_m_) of 10.8 ± 2.6 µg·mL^−1^ and a substrate inhibition constant (*K*_i_) of 73.7 ± 16 µg·mL^−1^ (Fig. S4b).

To identify conditions compatible with the GlyphoSense Chip, we evaluated ambient operational pH and temperature. A temperature of ~ 20 °C and a pH of 7.4 were selected, which allowed the enzyme to retain ~ 67% and ~ 73% of its optimal activity, respectively (Fig. S5a–b). These conditions ensure adequate GAT function without significantly compromising potential chip-based assay accuracy under field conditions^1^.

Since metal ions and structurally related chemicals can interfere with enzymatic activity^34,35^, we tested the effect of several ions (0.5 mM) in assay mixtures lacking EDTA (Fig. S5d). Among the ions tested, Al^3+^, Fe^2+^, and Zn^2+^ completely abolished GAT activity, while Ni^2+^ and Co^2+^ caused substantial reductions. As^3+^ and Mn^2+^ led to ~ 50% activity loss, whereas Ca^2+^ and Mg^2+^ had negligible effects. These findings highlight the importance of including metal chelators, such as EDTA, in assay formulations to mitigate interference from metal ions present in environmental samples^36^. If EDTA proves insufficient at standard concentrations (1 mM), its level may be increased in the assay to better minimize these effects.

We also assessed GAT activity against three structurally related glyphosate analogs: (aminomethyl)-phosphonic acid (AMPA), glufosinate, and glutamate (Fig. S5c). Of these, only AMPA, a primary glyphosate degradation product of similar toxicity, supported enzymatic activity, albeit at 8–10% of the rate observed with glyphosate as the substrate. Both glufosinate and glutamate showed negligible activity, indicating minimal cross-reactivity with GAT.

### GlyphoSense chip response and calibration

**Fig. 2:**
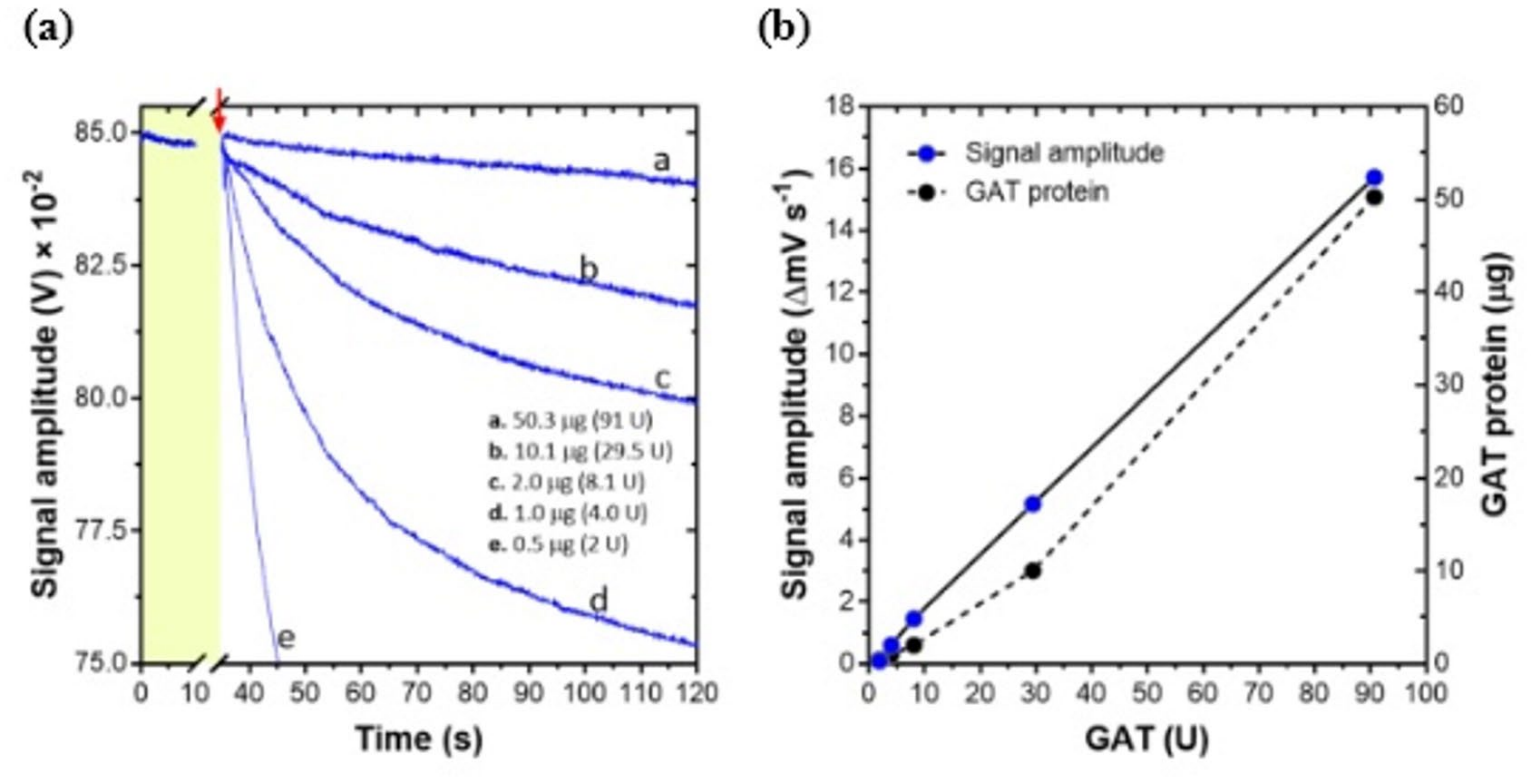
GlyphoSense Chip response to increasing GAT units. (**a**) Response of the GlyphoSense Chip at a fixed glyphosate concentration with increasing units of GAT. Responses were recorded in LabVIEW application over 120 s at a fixed concentration of glyphosate. The red arrow marks the point of AcCoA addition, indicating the initiation of the GlyphoSense Chip response to glyphosate in the applied sample. (**b**) Plot of GlyphoSense Chip signal (ΔmV s^−1^) with increasing GAT units. The measured signals reflect the GAT-catalyzed acetylation of glyphosate in the applied sample.

Glyphosate measurements using the GlyphoSense Chip were initiated by varying the amount of glyphosate *N*-acetyltransferase (GAT) added to the assay mixture (2, 4, 8.1, 29.5, and 90.7 units) at a fixed glyphosate concentration of 12.5 µg mL^−1^. A clear enzyme-dose response was observed, with a linear increase in the rate of change of signal amplitude (ΔmV s^−1^) corresponding to increasing GAT activity (U) or protein content (µg) in the assay mixture (Fig. 2a,b). Based on these results, a final concentration of ~ 3 U of GAT was selected as optimal for subsequent experiments. This concentration allowed reproducible capture and recording of signal amplitude rates at the lowest glyphosate concentrations tested, under the sensor operational setup for water sample measurements.

**Fig. 3:**
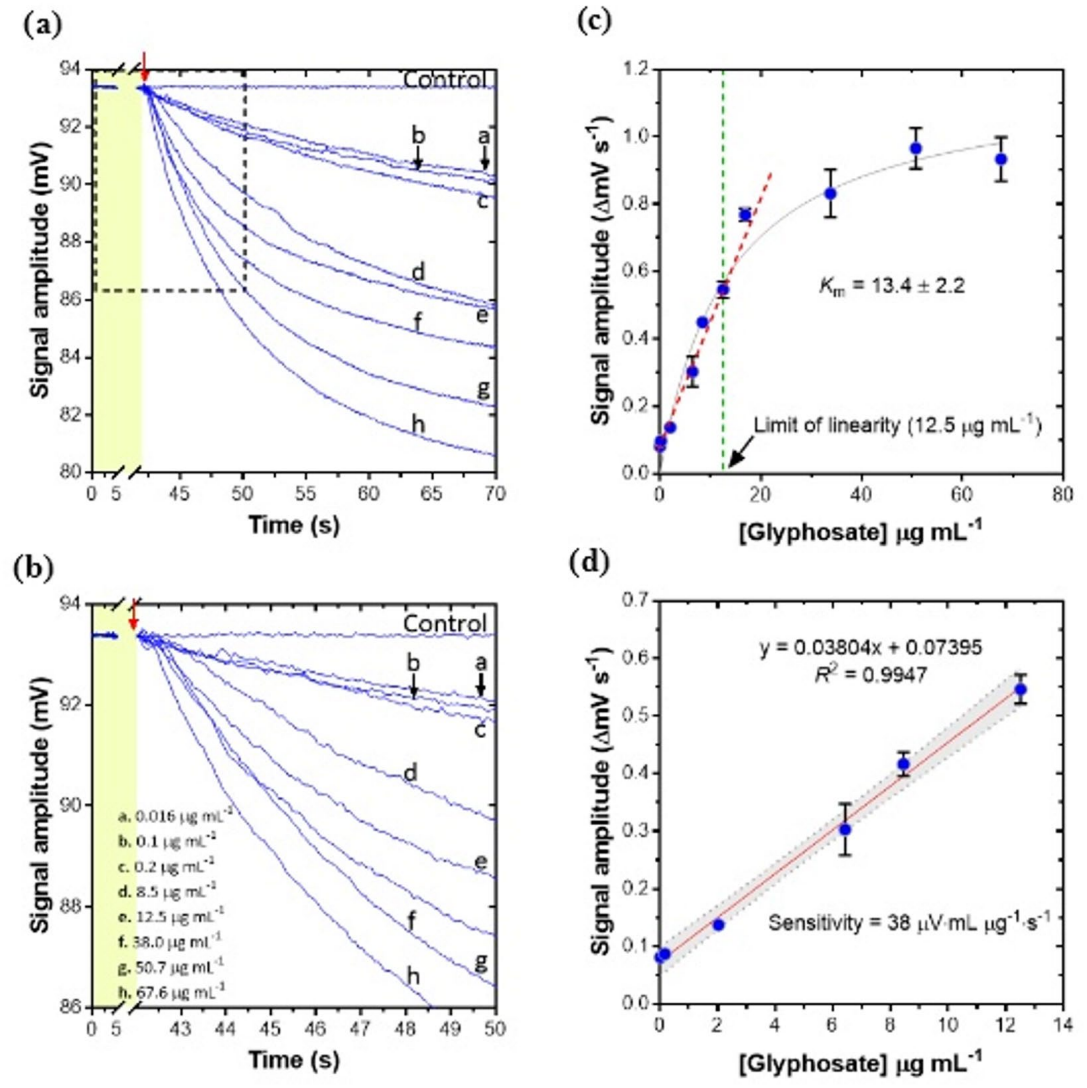
GlyphoSense Chip response to varying concentrations of glyphosate. (**a**) Calibration of the GlyphoSense Chip with glyphosate concentrations ranging from 0.016 to 67.6 µg mL^−1^. Responses were recorded in LabVIEW application over ~ 70 s at a fixed units of GAT (3.0 U). Red arrows indicate the initiation of the chip response; only signal transients caused by AcCoA addition were corrected. (**b**) Enlarged inset (dashed square) showing the linear portion of the kinetic curve (R^2^ > 0.98) used to calculate signal rates (ΔmV s^−1^). (**c**) Plot of chip response (ΔmV s^−1^) versus glyphosate concentration. The limit of linearity was 12.5 µg mL^−1^ (green dashed line), and the upper quantification value did not exceed the apparent *K*_m_ of GAT (13.4 ± 2.2 µg mL^−1^), determined by nonlinear regression (GraphPad Prism 10.4.1). (**d**) Calibration curve for glyphosate in water, with concentrations from 0.016 to 12.5 µg mL^−1^ fitted by linear regression (GraphPad Prism 10.4.1). Grey dashed lines represent the 95% confidence band. Data are means of independent experiments (*n* = 3); error bars represent SEM.

To define the linear operational calibration range of the biosensor, a broad range of glyphosate concentrations, spanning the *K*_m_ (10.8 ± 2.6 µg mL^−1^), was evaluated on the GlyphoSense Chip (0.016–67.63 µg mL^−1^) (Fig. 3). These concentrations were chosen to extend well beyond the substrate saturation levels established in preliminary off-chip spectrophotometric assays (Fig. S4). Each assay was recorded for at least 70 s to ensure accurate measurement of the initial signal amplitude rates (v₀ = ΔmV/Δt). The sensor response was strictly dependent on glyphosate concentration, initially showing a qualitative functional response followed by a saturation curve (Fig. 3a–b).

The sensor’s linearity and operational range were then assessed by constructing a calibration curve, plotting initial rates (first 10 s) versus nominal glyphosate concentrations from 0.016 to 67.63 µg mL^−1^ (Fig. 3c,d). The GlyphoSense Chip operates below substrate saturation (≤ 67.63 µg mL^−1^), and linearity was observed for concentrations between 0.016 and 12.5 µg mL^−1^. The upper quantification limit did not exceed the on-chip apparent *K*_m_ (13.4 ± 2.2 µg mL^−1^). For quantitative measurements, enzyme-based biosensor responses must remain linearly proportional to analyte concentration. In this case, linearity is maintained as long as glyphosate concentrations do not exceed the on-chip apparent *K*_m_, establishing an upper limit for accurate quantification^37^.

Linear regression analysis of initial rates versus glyphosate concentrations yielded the equation: ($y=0.0384x+0.07395$; ${R}^{2}=0.9947$). Residual analysis (Fig. 3d) allowed estimation of the standard error of the blank from the intercept (${s}_{a}$) using: ${s}_{a}={s}_{y/x}\sqrt{\frac{\sum{x}_{i}^{2}}{n\sum({x}_{i}-\overline{x}{)}^{2}}}$

where ${x}_{i}$ is the mean glyphosate concentration and${s}_{y/x}$ is the regression residual standard error. The limits of detection (LOD) and quantitation (LOQ) were determined from the standard error of the blank (${s}_{a}$ = 0.0093) using the formulas LOD = 3${s}_{a}$ and LOQ = 10${s}_{a}$^38,39^. Based on these calculations, the LOD was 0.0280 µg mL^−1^ and the LOQ was 0.0934 µg mL^−1^.

Regarding regulatory limits for glyphosate in drinking water, permissible concentrations vary worldwide from 0.0001 to 0.7 µg mL^−1^^40^. The UK Drinking Water Inspectorate (DWI) and the European Drinking Water Directive set a maximum of 0.0001 µg mL^−1^ for individual pesticides^10^. In regions where glyphosate levels exceed this limit, such as irrigation water, agricultural runoff, and livestock water, where permissible limits range from 0.2 to 4 µg mL^−1^^41^, the sensor may still be suitable for practical monitoring. Overall, the present GlyphoSense sensor is sensitive and “fit-for-purpose,” with detection and quantitation limits well below the US EPA guideline for drinking water (≤ 0.7 µg mL^−1^) and the Canadian interim guideline (≤ 0.280 µg mL^−1^)^41^. The developed GlyphoSense Chip represents a portable, handheld optical enzymatic biosensor for glyphosate detection, exhibiting detection performance and sensitivity comparable to other recently reported enzymatic biosensors employing diverse enzyme systems (Table 1), while offering a distinct operational advantage by providing a linear signal response for quantification in less than one minute.

**Table 1:** Examples of enzymatic biosensors reported for glyphosate detection.

Biosensing enzyme	Detection principle	Limit of detection (LOD)	Dynamic range	References
AChE	FET	6.4 × 10^− 8^ ppb	1.7 × 10^− 7^ − 1.7 × 10^− 3^ ppb	^47^
ACP	Amperometry	0.015 ppb	0.05 − 0.5 ppb	^48^
HRP	CV	1.70 ppb	0.25 − 14.0 ppb	^49^
Peroxidase	SWV	30 ppb	0.10 − 4.55 ppm	^50^
HRP	Electrochemical	5.44 ppb	16.9 ppb − 16.9 ppm	^51^
HRP	SWV	0.045 ppb	0.08 − 11 ppm	^52^
HRP	Amperometry	25 ppb	0.1 − 10 ppm	^25^
Tyrosinase	Amperometry	1.1 ppb	2.5 − 1.7 × 10^3^ ppb	^53^
Urease	Potentiometry	0.5 ppm	0.5 − 50 ppm	^30^
AChE, ChOx	ECL	8.5 × 10^− 2^ ppb	0.17 − 169 ppb	^54^
ALP, HRP	Amperometry	10 ppb	50 − 150 ppb	^55^
HRP	Amperometry	75 ppb	100 − 700 ppb	^56^
Peroxidase	AFS/Cantilevers	28 ppb	nd	^57^
GlyOx	Amperometry	0.5 ppm	1.7 − 44 ppm	^58^
GOX, HRP	Colorimetry	0.139 ppm	0.845 − 67.63 ppm	^59^
EPSPS-Ccg2 fusion protein	Colorimetry	8.45 ppb	8.45 ppb − 169.07 ppb	^60^
GAT	Colorimetry	28 ppb	0.016 − 12.5 ppm	This study

AChE: acetylcholinesterase ; ACP: acid phosphatase ; ALP: alkaline phosphatase; AFS: atomic force spectroscopy; ChOx: choline oxidase; CV: cyclic voltammetry; ECL: electrochemiluminescence; EPSPS: 5-enolpyruvylshikimate-3-phosphate synthase; FET: field-effect transistor ; GAT: glyphosate *N*-acetyltransferase; GlyOx: glycine oxidase; GOX: glyphosate oxidase; HRP: horseradish peroxidase; SWV: square wave voltammetry ; nd: not determined.

### GlyphoSense Chip validation and cross-validation

**Fig. 4:**
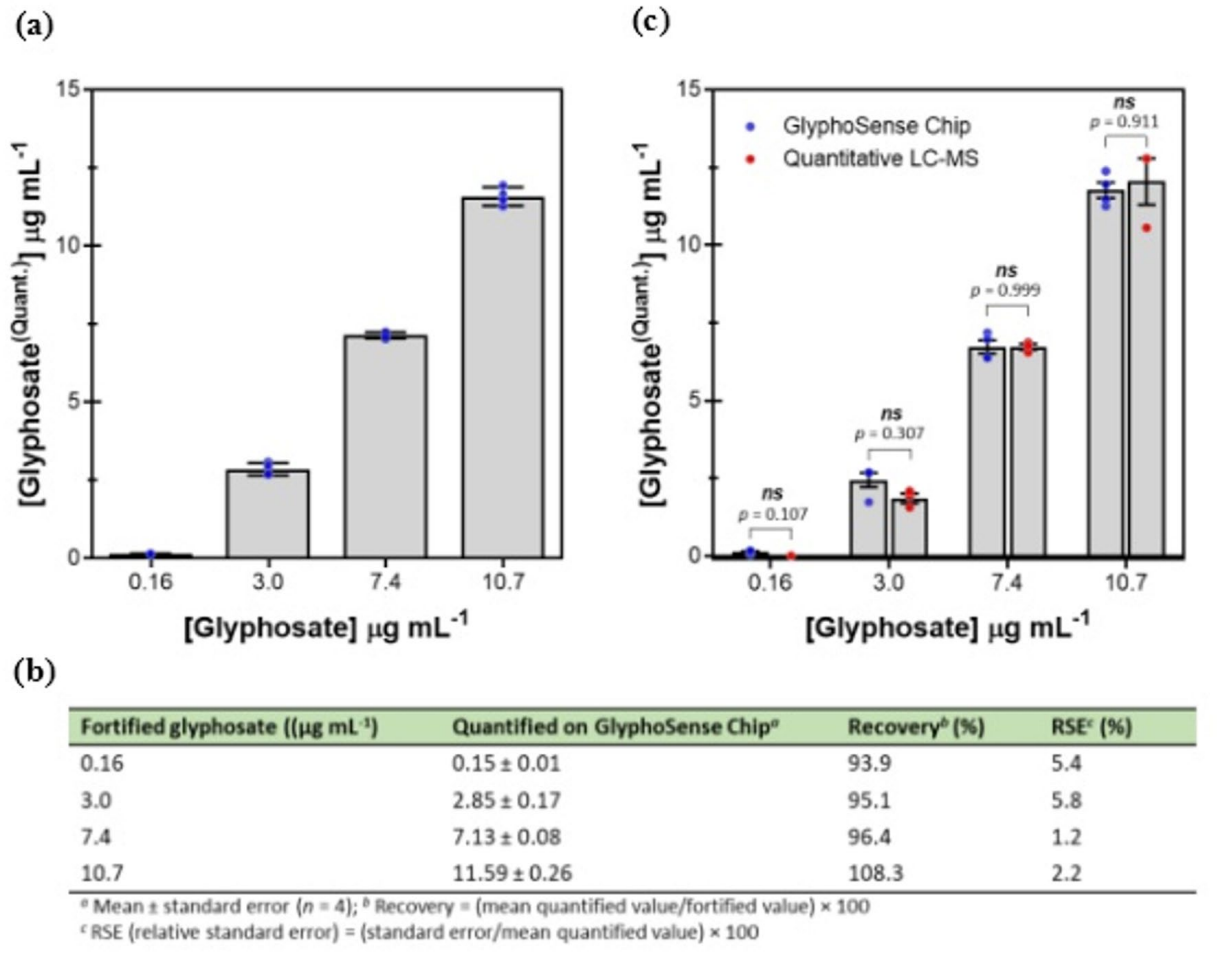
Validation of the GlyphoSense Chip. (**a**,**b**) Comparison of glyphosate concentrations in four known water samples (0.16, 3.0, 7.4, and 10.7 µg·mL^−1^) with values quantified on the GlyphoSense Chip. (**c**) Glyphosate concentrations measured on the GlyphoSense Chip (*n* = 4) were cross-validated by quantitative LC-MS analysis (*n* = 3; Table S1, Fig. S7). Statistical differences between the two quantification methods were assessed using multiple unpaired *t*-tests in GraphPad Prism 10.4.1. No significant difference was observed between the means of the two groups (*p* > 0.05; *ns* denotes not significant).

To confirm the analytical validation of the GlyphoSense Chip, regression analysis of variance (ANOVA) was performed on measured concentration values to evaluate the significance of GlyphoSense Chip-determined glyphosate levels. No statistically significant differences were observed between four fortified water samples containing known glyphosate concentrations and the concentrations quantified by the GlyphoSense Chip (*p* > 0.05) (Fig. 4a–b). For inter-day cross-validation, GlyphoSense Chip measurements were compared against an independent gold-standard analytical method. Glyphosate concentrations quantified by the GlyphoSense Chip were further analyzed using liquid chromatography-mass spectrometry (LC-MS) (Table S1). Statistical comparisons of the two datasets using multiple unpaired *t-*tests showed no significant differences (*p* > 0.05) (Fig. 4c), confirming the strong agreement of GlyphoSense Chip measurements with LC-MS, a widely accepted reference method for glyphosate detection^42^. To further evaluate the performance of the GlyphoSense Chip, its accuracy and precision were tested across the device’s dynamic range using fortified water samples containing low, medium, and high glyphosate concentrations (0.16, 3.0, 7.4, and 10.7 µg mL^−1^). Accuracy was expressed as percentage recovery, calculated from the mean of replicate measurements^43,44^, while precision was determined by intra-day repeatability and expressed as the relative standard error (RSE) from a statistically sufficient number (*n* = 4) of replicates (Fig. 4b). Both parameters were within acceptable validation limits, confirming the robustness of the GlyphoSense Chip.

### GlyphoSense Chip selectivity

A common challenge for biosensors is reduced selectivity when analyzing environmental samples of varying matrix complexity. Unlike separation-based technologies such as chromatography or mass spectrometry, chemical sensors often show diminished performance because of potential interference from diverse chemical species present in real samples. Such interferences can compromise glyphosate quantification. To evaluate the selectivity of the GlyphoSense Chip, we tested several compounds structurally related to glyphosate: AMPA (aminomethylphosphonic acid), glufosinate, glutamate, and the commercial formulation Roundup. Each compound was prepared at the same concentration as glyphosate (8.45 µg mL^−1^, 50 µmol L^−1^) to allow direct comparison. Since commercial formulations like Roundup contain surfactants and additives such as polyethoxylated tallow amine (POEA), which can influence sensor performance^45^, we compared pure glyphosate with Roundup to assess potential matrix effects. We also tested AMPA, the primary degradation product of glyphosate, to determine whether it interferes with GlyphoSense Chip response.

**Fig. 5:**
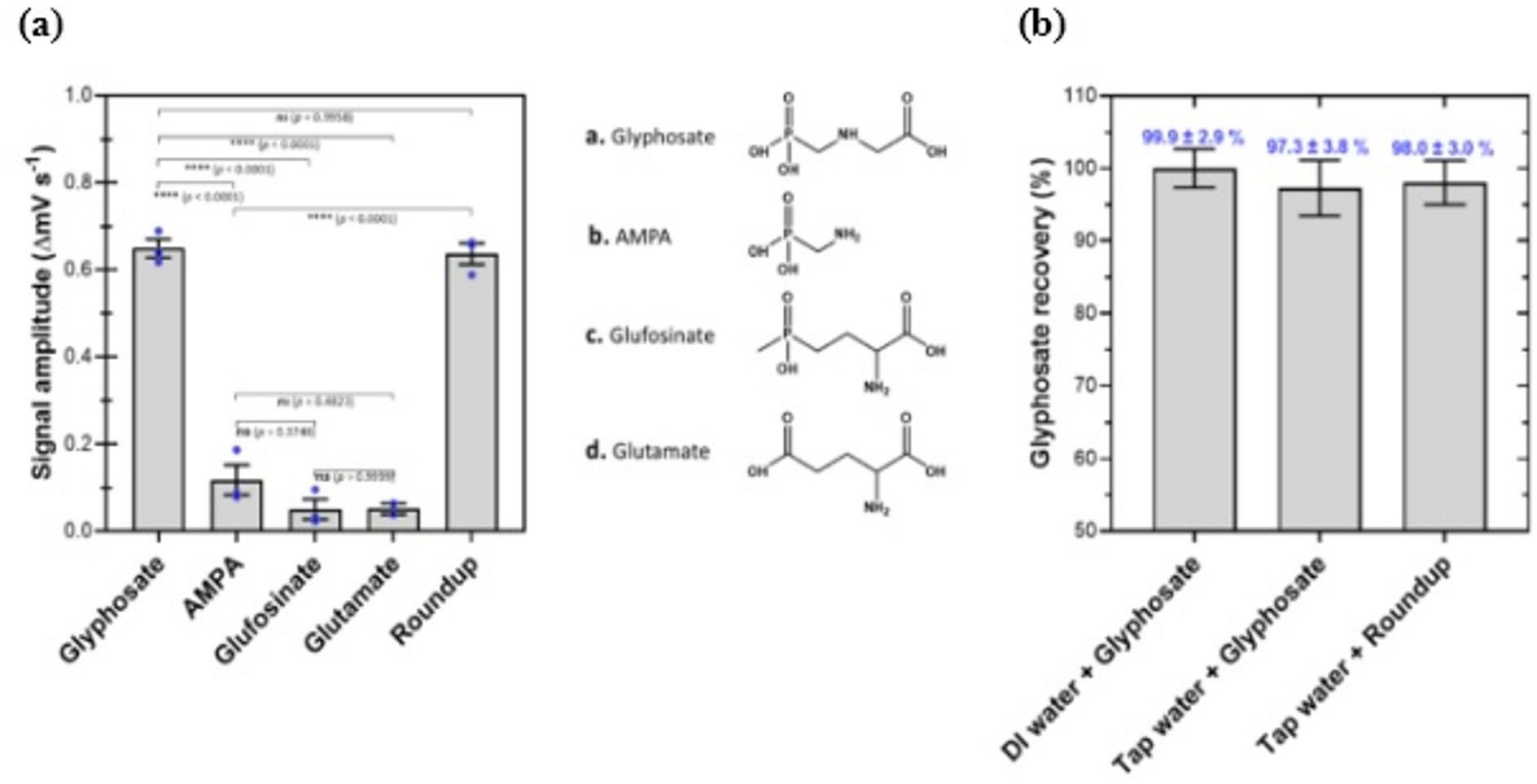
GlyphoSense Chip response to glyphosate and structural analogs. (**a**) Response of the GlyphoSense Chip to glyphosate, a commercial Roundup formulation, and structural analogs [aminomethylphosphonic acid (AMPA), glufosinate (ammonium salt), and glutamate] in tap water. All structural analogs were tested at concentrations equal to glyphosate. (**b**) Matrix effects were assessed by spiking local tap water with glyphosate or the Roundup formulation at equivalent glyphosate concentrations (1.0–8.45 µg mL^−1^), and percentage recovery was calculated. Data represent means of independent measurements (*n* = 3), with error bars indicating SEM.

The results showed that glyphosate in Roundup elicited nearly identical response to pure glyphosate (*p* > 0.05), indicating that formulation additives did not hinder detection. In contrast, glufosinate and glutamate produced only minor responses, suggesting minimal cross-reactivity. AMPA generated a low response relative to glyphosate, confirming that it did not significantly interfere with sensor measurements (Fig. 5a).

### Water samples

The GlyphoSense Chip was evaluated for matrix interference using locally collected tap water spiked with 0.5 µg mL^−1^ glyphosate and 8.45 µg mL^−1^ Roundup. Under optimized validation conditions, the chip produced quantitative responses comparable to those obtained with deionized water (Fig. 5b). Glyphosate recoveries were 97.3 ± 3.8% and 98.0 ± 3.0% for glyphosate and Roundup, respectively, confirming both accuracy and reproducibility. These findings demonstrate that the GlyphoSense Chip performs reliably in real water samples without requiring extensive pre-treatment, underscoring its robustness and applicability for routine monitoring of glyphosate contamination of drinking water resources^46^.

## Conclusions

Glyphosate is one of the most widely used herbicides worldwide, which has been implicated in carcinogenesis. The lack of deployable on-site screening devices has put a limitation on routine monitoring of drinking water resources for this potential carcinogenic contaminant. The GlyphoSense Chip platform demonstrated here is able to detect glyphosate in contaminated water down to 0.028 µg mL^−1^. The platform’s performance was cross-validated using a gold standard technique, quantitative LC-MS, and it delivers sensitivity and accuracy comparable to this established method. The selectivity and sensitivity of the GlyphoSense Chip derive from the use of a cloned, expressed, and in-house purified genetically evolved *N*-acetyltransferase with a high specificity for glyphosate. The limit of detection achieved by the GlyphoSense Chip is lower than the permissible limits of glyphosate in water allowed by most regulatory authorities worldwide. This prototype also benefits from a user-friendly interface and its portability and field-ready functionality. Thus, the GlyphoSense Chip’s validated performance, portability, and ease of use establish it as a powerful tool for water safety monitoring, particularly in contexts requiring real-time detection or where glyphosate use is subject to increasing regulatory scrutiny.

## Supplementary Information

Below is the link to the electronic supplementary material.

Supplementary Material 1

## Data Availability

The raw data generated and/or analyzed during the current study available from the corresponding author on reasonable request.
