# Supplementary material for: Rapid detection and quantification of glyphosate in water using a handheld portable biosensor
Source: Sci Rep. 2026 Apr 11;16:16913. doi: 10.1038/s41598-026-44827-4 (PMC13230554; doi:10.1038/s41598-026-44827-4)
Supplement: Supplementary file 1 — Supplementary Material 1 [file 41598_2026_44827_MOESM1_ESM.docx]

**Supplementary Information**

**Rapid detection and quantification of glyphosate in water using a handheld portable biosensor**

Andreea Stroia^a^, Boon Chong Cheah^b^, Samadhan B. Patil^b,^ ^c, d^, Valerio F. Annese^b^, Reynard Spiess^e^, Christoph Busche^f^, David R. S. Cumming^b^, Michael P. Barrett^a,^*, Dharmendra S. Dheeman^a, c,^ *

^a^ *Wellcome Centre for Integrative Parasitology, Institute of Infection, Immunity and Inflammation, University of Glasgow, Glasgow G12 8TA, UK United Kingdom*

^b^ *Electronics and Nanoscale Engineering, James Watt School of Engineering, University of Glasgow, Glasgow, G12 8LT, United Kingdom*

^c^ *School of Physics, Engineering and Technology, University of York, Heslington, York,*

*YO10 5DD*, *United Kingdom*

^d^ *York Biomedical Research Institute, University of York, Heslington, York YO10 5NG,*

*United Kingdom*

^e^ *Manchester Institute of Biotechnology, School of Chemistry, University of Manchester, 131 Princes Street, Manchester M1 7DN, United Kingdom*

^f^ *School of Chemistry, University of Glasgow, Glasgow G12 8QQ, United Kingdom*

* **Corresponding authors:**

**Dharmendra S. Dheeman**, E-mail:[dharmendra.dheeman@york.ac.uk](mailto:dharmendra.dheeman@york.ac.uk)

**Michael P. Barrett**, E-mail: [michael.barrett@glasgow.ac.uk](mailto:michael.barrett@glasgow.ac.uk)

# **Synthetic GAT gene**

The synthetic *gat* gene [3,4] was obtained from GenScript (Hong Kong) with codons optimized for *Escherichia coli* expression and flanked by NdeI (5′) and BamHI (3′) restriction sites (Fig. S1). It was supplied subcloned into the EcoRV site of the non-expression vector pUC57-Kan.

**R11 *gat*** ATGATAGAGGTAAAACCGATTAACGCAGAGGATACCTATGACCTAAGGCATAGAGTCCTC

**Synthetic *gat*** ATGATCGAGGTGAAGCCGATTAACGCGGAAGACACCTACGATCTGCGTCACCGTGTTCTG

***** ***** ** *********** ** ** ***** ** ** * ** * ** **

**R11 *gat*** AGACCAAACCAGCCGATAGAAGCGTGTATGTTTGAAAGCGATTTAACGCGTAGTGCATTT

**Synthetic *gat*** CGTCCGAACCAGCCGATCGAGGCGTGCATGTTCGAAAGCGACCTGACCCGTAGCGCGTTC

* ** *********** ** ***** ***** ******** * ** ***** ** **

**R11 *gat*** CACTTAGGCGGCTTCTACGGGGGCAAACTGATTTCCGTCGCTTCATTCCACCAGGCCGAG

**Synthetic *gat*** CACCTGGGTGGCTTTTATGGTGGCAAACTGATTAGCGTGGCGAGCTTTCACCAGGCGGAG

*** * ** ***** ** ** ************ *** ** ** ******** ***

**R11 *gat*** CACTCGGAACTTCAAGGCAAGAAACAGTACCAGCTTCGAGGTGTGGCTACCTTGGAAGGT

**Synthetic *gat*** CACAGCGAACTGCAAGGTAAGAAACAGTACCAACTGCGTGGTGTGGCGACCCTGGAGGGC

*** ***** ***** ************** ** ** ******** *** **** **

**R11 *gat*** TATCGTGAGCAGAAGGCGGGTTCCAGTCTAGTTAAACACGCTGAAGAAATTCTACGTAAG

**Synthetic *gat*** TATCGTGAACAGAAGGCGGGCAGCAGCCTGGTTAAACACGCGGAGGAAATCCTGCGTAAG

******** *********** *** ** *********** ** ***** ** ******

**R11 *gat*** AGGGGGGCGGACATGATTTGGTGTAATGCGCGGACATCTGCCTCAGGCTACTACAGAAAG

**Synthetic *gat*** CGTGGTGCGGACATGATTTGGTGCAACGCGCGTACCAGCGCGAGCGGTTACTATCGTAAA

* ** ***************** ** ***** ** ** ** ***** * **

**R11 *gat*** TTAGGCTTCAGCGAGCAGGGAGAGGTATTCGACACGCCGCCAGTAGGACCTCACATCCTG

**Synthetic *gat*** CTGGGTTTCAGCGAGCAAGGCGAAGTGTTTGATACCCCGCCGGTTGGTCCGCACATCCTG

* ** *********** ** ** ** ** ** ** ***** ** ** ** *********

**R11 *gat*** ATGTATAAAAGGATCACATAA

**Synthetic *gat*** ATGTACAAGCGTATTACCTAA

***** ** * ** ** ***

**Fig. S1. Sequence of the R11 *gat* gene (GenBank accession no. AY597418) and its codon-optimized synthetic version for expression in *Escherichia coli*.** Conserved nucleotides are marked with asterisks (*).

# **GAT expression and purification**

The expression plasmid pET14b-gat was transformed into *E. coli* BL21(DE3). Cultures were grown in LB medium containing ampicillin (100 µg mL^−1^) at 37 °C, 200 rpm until mid-log phase (OD600 ≈ 0.6). Protein expression was induced with 0.3 mM IPTG, and cultures were incubated for an additional 3 h. Cells were harvested by centrifugation (6000 rpm, 15 min, 4 °C), washed, and resuspended in binding buffer (50 mM NaH_2_PO_4_, 300 mM NaCl, pH 7.4; 20 mL per gram of wet cells).

Cell lysis was performed by sonication (5 cycles, 18 μm amplitude, 10 s pulse/20 s pause; Soniprep 150, MSE Ltd., UK) in the presence of protease inhibitors (cOmplete Mini, EDTA-free, Roche). Cell debris was removed by ultracentrifugation (35,000 rpm, 30 min), and the clarified lysate was applied to a 1 mL His GraviTrap Ni-affinity column (GE Healthcare, UK) pre-equilibrated with binding buffer. After washing with 200 column volumes of wash buffer (50 mM NaH_2_PO_4_, 300 mM NaCl, 20 mM imidazole, pH 7.4), GAT (Fig. S2) was eluted with 3 column volumes of elution buffer (50 mM NaH_2_PO_4_, 300 mM NaCl, 250 mM imidazole, pH 7.4).

The eluted protein was concentrated approximately 10-fold (to 0.25 mL) using a 10 kDa cut-off centrifugal filter (Amicon Ultra-15, Millipore) and washed three times with TBS (50 mM Tris-HCl, pH 7.4, 150 mM NaCl) to remove imidazole. For further purification and determination of the oligomeric state, the sample was subjected to gel filtration on a Superdex 75 10/300 GL column (GE Healthcare) at 4 °C, pre-equilibrated with TBS. Protein elution was monitored at 280 nm, with molecular mass standards indicated above the chromatogram peaks (Fig. S3).

Fractions from both the affinity (lane 1) and gel-filtration (lane 2) steps were analyzed by SDS-PAGE (Fig. S3b), with molecular mass markers shown in lane 3 (kDa).

**R11 GAT** --------------------MIEVKPINAEDTYDLRHRVLRPNQPIEACMFESDLTRSAF

**GAT** MGSSHHHHHHSSGLVPRGSHMIEVKPINAEDTYDLRHRVLRPNQPIEACMFESDLTRSAF

****************************************

**R11 GAT** HLGGFYGGKLISVASFHQAEHSELQGKKQYQLRGVATLEGYREQKAGSSLVKHAEEILRK

**GAT** HLGGFYGGKLISVASFHQAEHSELQGKKQYQLRGVATLEGYREQKAGSSLVKHAEEILRK

************************************************************

**R11 GAT** RGADMIWCNARTSASGYYRKLGFSEQGEVFDTPPVGPHILMYKRIT

**GAT** RGADMIWCNARTSASGYYRKLGFSEQGEVFDTPPVGPHILMYKRIT

**********************************************

**Fig. S2. Amino acid sequence of recombinant GAT.** The sequence represents the synthetic gat gene cloned into the NdeI and BamHI sites of the pET15b vector and expressed in *E. coli* BL21(DE3). The alignment highlights differences between R11 GAT (AAU04689) and the recombinant GAT used in this study. Vector-derived residues are underlined, and conserved amino acids are indicated by asterisks (*)

**(a)**

**(b)**

**Fig. S3. Size-exclusion chromatography and SDS-PAGE analysis of GAT.** (a) Purified GAT from a Ni-NTA affinity column was further separated on a Superdex 75 10/300 GL column. Elution profiles of GAT (navy) and protein standards (light brown) indicate that GAT exists as a monomer. (b) SDS-PAGE analysis of GAT (18.7 kDa) after Ni-NTA purification (lane 1) and gel filtration (lane 2). Lane 3 shows molecular weight markers in kDa (Bio-Rad Precision Plus Protein Dual Xtra Standards).

# **Off-chip GAT kinetics**

The kinetic parameters of glyphosate for purified GAT were determined using a continuous spectrophotometric assay. The assay monitored the hydrolysis of 0.2 mM acetyl-CoA (AcCoA) and the subsequent acetylation of glyphosate (4.2–101.4 µg mL^−1^) via the reaction of free sulfhydryl groups with 0.32 mM 5,5’-dithiobis-2-nitrobenzoate (DTNB) [1,2]. Reactions (250 µL) were performed at 25 °C in 20 mM Tris-HCl (pH 7.4) containing 1 mM EDTA, initiated by adding appropriately diluted GAT, and monitored at 412 nm using a Shimadzu UV-Vis spectrophotometer. Test reactions were compared with parallel no-enzyme and no-substrate controls. A decrease in GAT activity was observed at high glyphosate concentrations (>74 µg mL^−1^). Initial reaction rates were used to calculate the *K*_m_ for glyphosate (4.2–338.1 µg mL^−1^) using a substrate inhibition model in GraphPad Prism 10.4.1 (Fig. S4).

**
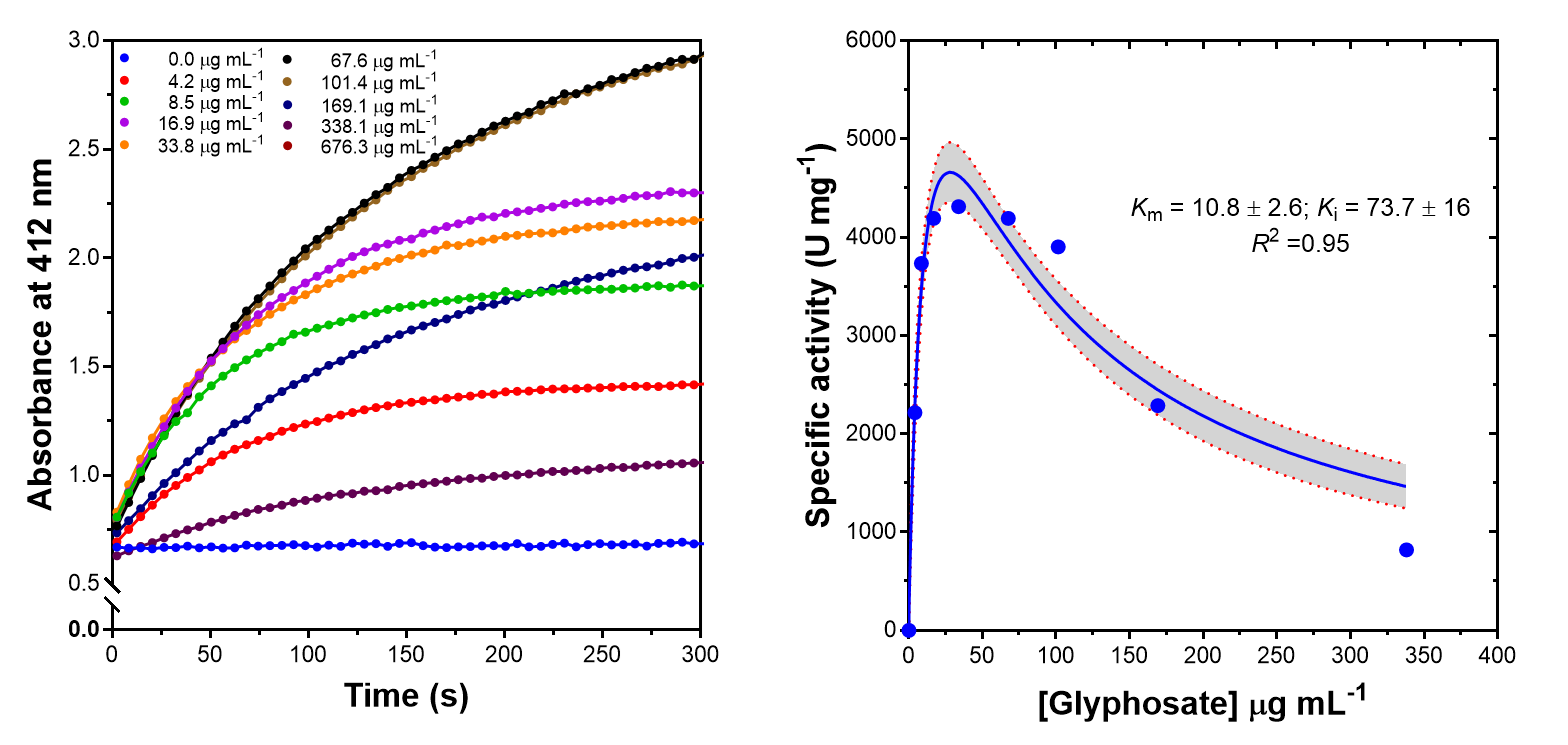
**

**(a)**

**(b)**

**Fig. S4. Off-chip GAT kinetics.** (a) Initial reaction rates measured over a glyphosate concentration range of 4.2–338.1 µg mL^−1^. (b) Off-chip kinetic parameters, *K*_m_ and *K*_i_, were determined by fitting the data to the non-linear regression equation $v=V_{max}\times\left[ S \right]/(K_{m}+\left[ S \right]*(1+[S]/K_{i}$ using GraphPad Prism 10.4.1.

# **Off-chip GAT activity**

GAT activity was measured across a temperature range of 10–50°C and pH 5.0–8.5 using a continuous spectrophotometric assay (Fig. S5a-b). Substrate specificity was determined by replacing glyphosate with equimolar concentrations (50 µM) of its structural analogs, aminomethylphosphonic acid (AMPA), glufosinate (phosphinothricin), or glutamate, under standard assay conditions (Fig. S5c). The impact of various ions was assessed by substituting 1 mM EDTA in the assay with 0.5 mM of each ion (Fig. S5d). Enzyme activity was calculated from initial reaction rates, with no-enzyme and no-substrate controls run in parallel to correct for background.

**(a)**

**(b)**

**(c)**

**(d)**


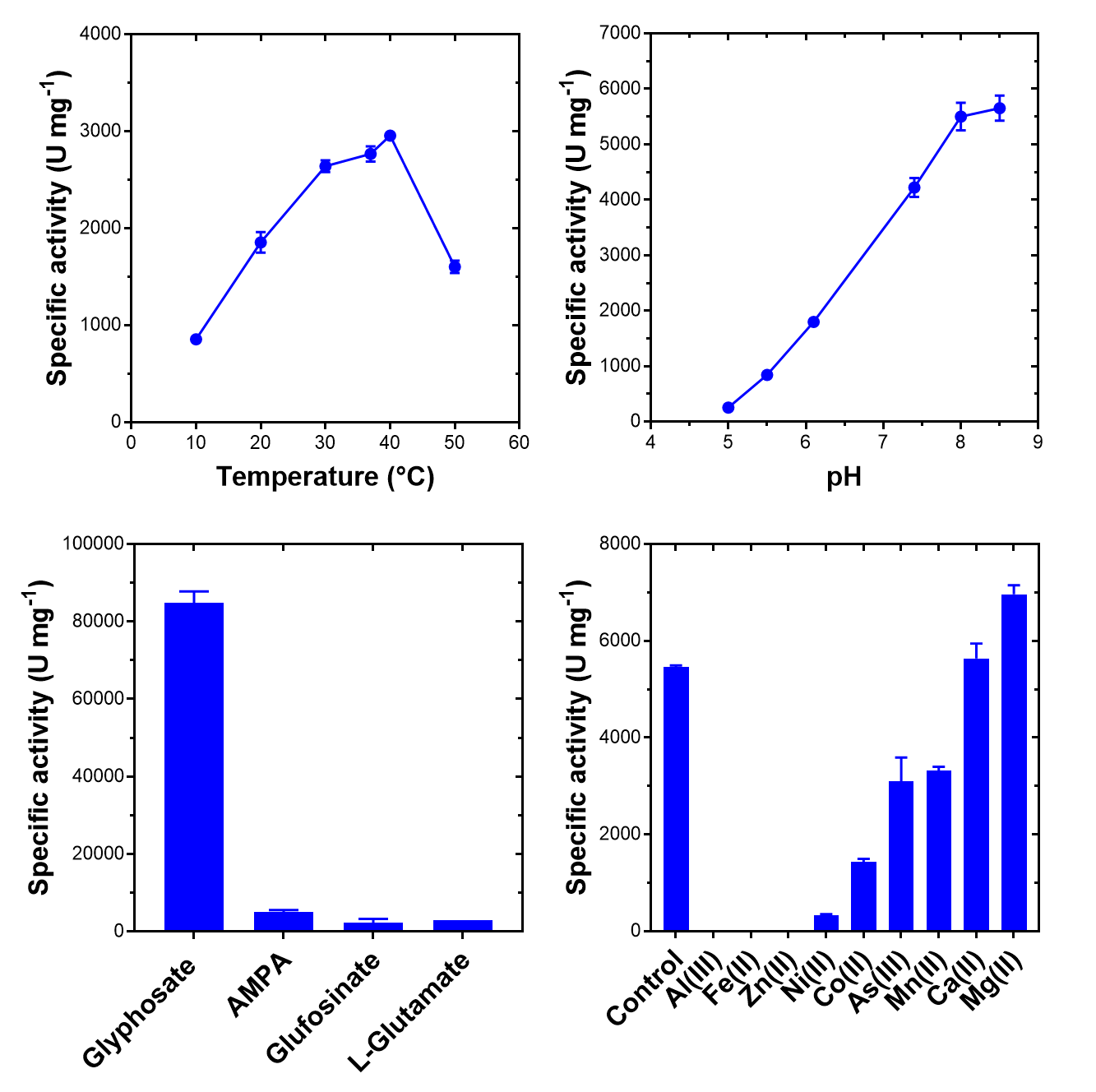


**Fig. S5. Off-chip GAT activity.** (a–b) Effect of temperature and pH on GAT activity. Enzyme activity was measured at temperatures ranging from 10 to 50 °C and at pH values from 5.0 to 8.5. (c) Substrate specificity of GAT. Activity was assessed using 50 µM of various structural analogs in place of glyphosate in the standard assay. (d) Effect of ions on GAT activity. The standard assay containing 1 mM EDTA was modified by replacing EDTA with 0.5 mM of different ions.

# **GlyphoSense Chip assay**

Glyphosate detection in the GlyphoSense Chip assay was based on the enzymatic activity of GAT, which catalyzes the transfer of an acetyl group from acetyl-CoA to the secondary amine of glyphosate, producing N-acetylglyphosate and releasing coenzyme A (CoA-SH) by cleavage of the thioester bond. The liberated CoA-SH subsequently reacts with 5,5′-dithiobis(2-nitrobenzoic acid) (DTNB) in a coupled chromogenic reaction, forming the yellow anion 2-nitro-5-thiobenzoate (TNB), which can be quantified colorimetrically[3,4].


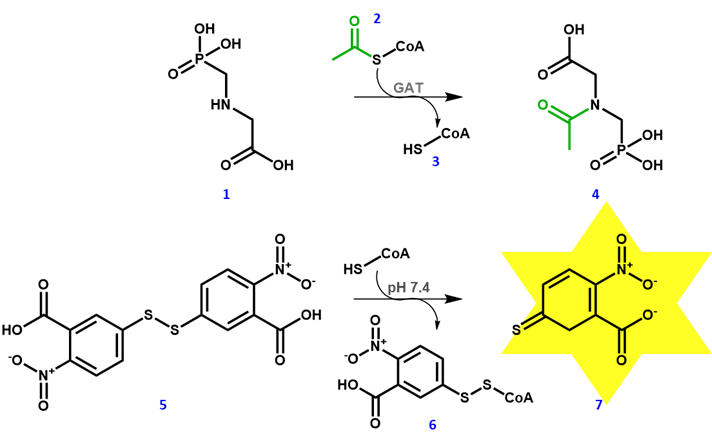


**Fig. S6. GAT-mediated glyphosate detection in the GlyphoSense Chip assay.** The upper panel shows acetylation of glyphosate by GAT, forming N-acetylglyphosate and CoA-SH; the lower panel shows the DTNB-mediated reaction of CoA-SH that generates the yellow TNB chromophore for colorimetric detection. Reaction components are indicated numerically: (1) glyphosate, (2) acetyl-CoA, (3) CoA-SH, (4) N-acetylglyphosate, (5) DTNB, (6) mixed-disulfide byproduct, and (7) TNB.

# **GlyphoSense Chip cross-validation by LC-MS**


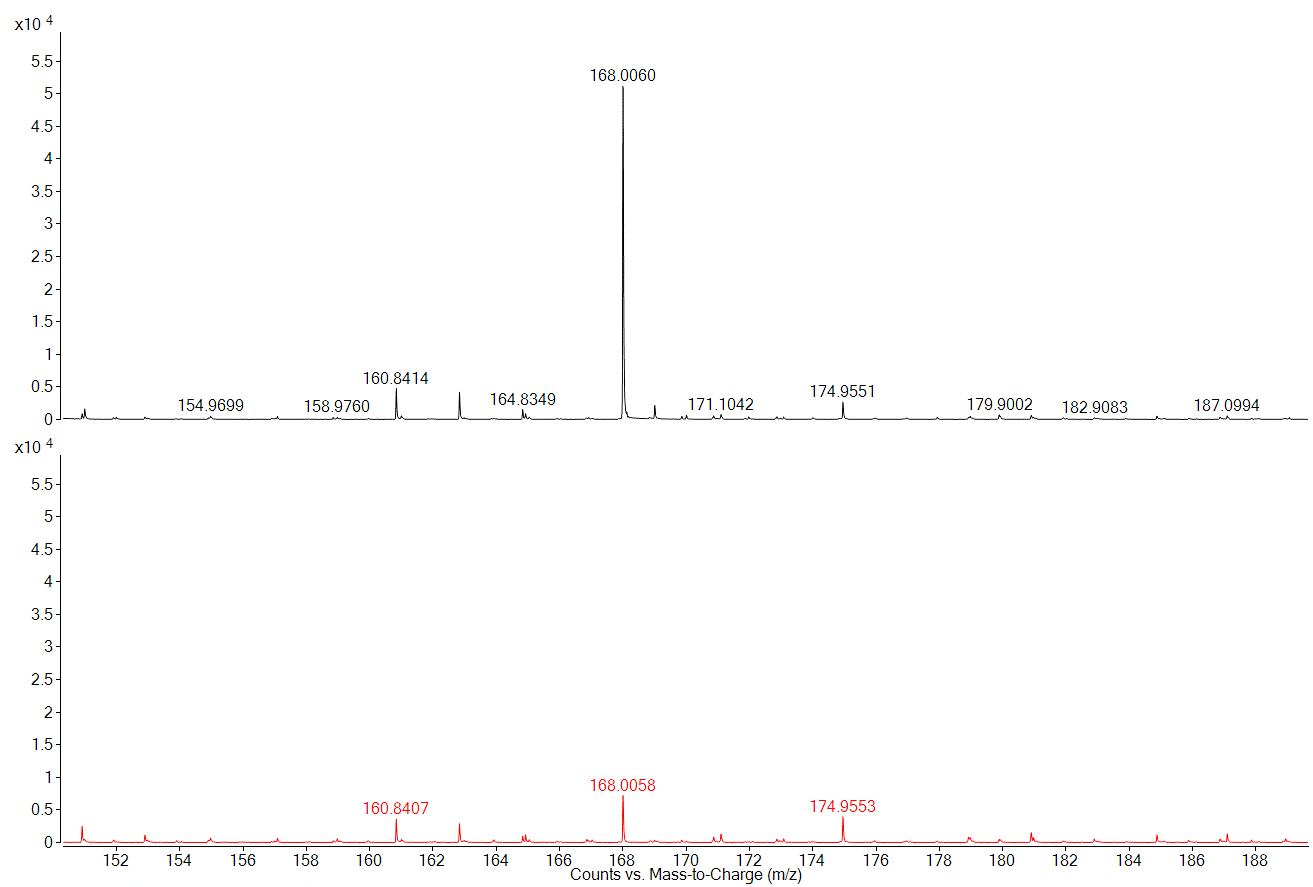
The GlyphoSense Chip was cross-validated using an independent LC-MS method for glyphosate quantification [5]. Triplicate glyphosate samples in deionized water were analyzed via flow injection. Five microliters of each sample were injected using an Agilent 1200 Series HPLC (Agilent Technologies, Santa Clara, USA) with a mobile phase consisting of 50% acetonitrile, 50% water, and 0.1% formic acid (v/v) at a flow rate of 0.3 mL min^−1^. Detection was performed on an Agilent 6520 Q-TOF mass spectrometer operating in ESI negative mode, and data were processed using Agilent MassHunter software. The integrated peak area of the dominant [M−H]⁻ ion at m/z 168.0067 (Fig. S6) was used to generate a calibration curve from glyphosate standards over the concentration range 0.002–169.1 µg mL^−1^.

**Fig. S7. Cross-validation of the GlyphoSense Chip using LC-MS.** (a) Representative spectra from triplicate LC-MS runs. Top: glyphosate standard (33.8 µg mL^−1^). Bottom: glyphosate test sample (3.0 µg mL^−1^ in tap water) quantified by the GlyphoSense Chip. (b) LC-MS calibration curve for glyphosate quantification, generated from triplicate injections of standards in water over a concentration range of 0.002–169.1 µg mL^−1^. To benchmark the GlyphoSense Chip, samples quantified by the chip were subsequently analysed by LC-MS, and concentrations were determined from peak areas (Table S1) using the calibration curve.

**Table S1.** Cross-validation of GlyphoSense Chip glyphosate measurements against LC–MS

| **Glyphosate (µg mL^-1^)** | | | **MS Peak Area** | |
| --- | --- | --- | --- | --- |
| 0.16 | 4.74×10^3^ | 3.65×10^3^ | | 4.74×10^3^ |
| 3.0 | 4.06×10^5^ | 5.02×10^5^ | | 5.42×10^5^ |
| 7.4 | 1.78×10^6^ | 1.70×10^6^ | | 1.76×10^6^ |
| 10.7 | 2.74×10^6^ | 3.33×10^6^ | | 3.32×10^6^ |

**References**

1. Castle, L. A. *et al.* Discovery and directed evolution of a glyphosate tolerance gene. *Science (1979)* **304,** 1151–1154 (2004).
2. Siehl, D. L., Castle, L. A., Gorton, R. & Keenan, R. J. The molecular basis of glyphosate resistance by an optimized microbial acetyltransferase. *Journal of Biological Chemistry* **282,** 11446–11455 (2007).
3. Thompson, C. J. *et al.* Characterization of the herbicide-resistance gene bar from Streptomyces hygroscopicus. *EMBO J* **6,** 2519–2523 (1987).
4. Wehrmann, A., Van Vliet, A., Opsomer, C., Botterman, J. & Schulz, A. The Similarities of Bar and Pat Gene Products make them Equally Applicable for Plant Engineers. *Nat Biotechnol* **14,** 1274–1278 (1996).

5. Taylor, J. K. Quality Assurance of Chemical Measurements. *Anal Chem* **53,** 1588A–1596A (1981).
